# From ownership to custodianship of tumor biopsy tissue in genomic testing: a mixed methods study of patient views

**DOI:** 10.1093/oncolo/oyae074

**Published:** 2024-05-07

**Authors:** Megan C Best, Phyllis Butow, Jacqueline Savard, Ainsley J Newson, Rachel Campbell, Sabina Vatter, Christine E Napier, Nicci Bartley, Katherine Tucker, Mandy L Ballinger, David M Thomas, the PiGeOn Project, Megan C Best, Phyllis Butow, Jacqueline Savard, Ainsley J Newson, Rachel Campbell, Sabina Vatter, Christine E Napier, Nicci Bartley, Katherine Tucker, Mandy L Ballinger, David M Thomas, Ilona Juraskova, Ainsley Newson, Timothy Schlub, Grace Davies, Bettina Meiser, David Goldstein

**Affiliations:** Institute for Ethics and Society, The University of Notre Dame Australia, Broadway, NSW, Australia; Psycho-Oncology Co-operative Research Group, School of Psychology, University of Sydney, Sydney, NSW, Australia; Faculty of Health, School of Medicine, Deakin University, Melbourne, VIC, Australia; Sydney Health Ethics, Faculty of Medicine and Health, Sydney School of Public Health, University of Sydney, Sydney, NSW, Australia; Faculty of Science, School of Psychology, University of Sydney, Sydney, NSW, Australia; Psycho-Oncology Co-operative Research Group, School of Psychology, University of Sydney, Sydney, NSW, Australia; Garvan Institute of Medical Research, Sydney, NSW, Australia; Psycho-Oncology Co-operative Research Group, School of Psychology, University of Sydney, Sydney, NSW, Australia; Hereditary Cancer Centre, Prince of Wales Medical School, Prince of Wales Hospital, University of New South Wales, Randwick, NSW, Australia; Garvan Institute of Medical Research, St Vincent’s Clinical School, UNSW, Sydney, NSW, Australia; Garvan Institute of Medical Research, St Vincent’s Clinical School, UNSW, Sydney, NSW, Australia

**Keywords:** genomics, tumor biopsy, medical ethics, patient perspectives

## Abstract

Tumor mutation profiling (MP) is often conducted on tissue from biopsies conducted for clinical purposes (diagnostic tissue). We aimed to explore the views of patients with cancer on who should own tumor biopsy tissue, pay for its storage, and decide on its future use; and determine their attitudes to and predictors of undergoing additional biopsies if required for research purposes. In this mixed methods, cross-sectional study, patients with advanced solid cancers enrolled in the Molecular Screening and Therapeutics Program (*n* = 397) completed a questionnaire prior to undergoing MP (*n* = 356/397). A subset (*n* = 23) also completed a qualitative interview. Fifty percent of participants believed they and/or relatives should own and control access to diagnostic tissue. Most (65.5%) believed the government should pay for tissue preparation. Qualitative themes included (1) custodianship of diagnostic tissue, (2) changing value of tissue across time and between cultures, (3) equity regarding payment, and (4) cost-benefit considerations in deciding on additional biopsies. Policy and regulation should consider patient perspectives. Extension of publicly funded health care to include tissue retrieval for clinical trials should be considered.

**Implications for Practice**

This article provides internationally novel data on the views of patients with advanced cancer regarding who should own, control use of, pay for, and make decisions about tumor biopsy tissue collected for clinical purposes but also needed for genome research. The legal and ethical basis for resolving these questions remains markedly unclear. Results suggest that patients’ views on a continuing relationship with their excised tissue should be respected. Transparency and ongoing communication with patients, if desired, regarding how tissue will be used, is important. Larger tissue samples at excision should be considered, alongside government funding for research access to clinical samples.

## Introduction

Tumor mutation profiling (MP) is increasingly used to guide therapeutic care in oncology. However, knowledge of the meaning of genetic variation is still accruing and MP is still commonly undertaken within the research context. This often requires obtaining access to samples previously excised during clinical care. Tissue samples are stored in a variety of settings, including by both publicly and privately funded entities. Consequently, there is confusion amongst patients, physicians, researchers, and institutions regarding who owns and can access previously excised tissue for MP, as well as who should pay for any processing involved.

Human tissue is usually excised for diagnostic or treatment purposes. All sampled and processed diagnostic tissue must be retained for future diagnostic use should it become needed, for a minimum set period (usually around 7-20 years, dependent on local statutory and laboratory licensing and accreditation requirements), in the local pathology department archives.^1^ They are stored in the form of formalin-fixed, paraffin-embedded tissue blocks. As this tissue is a limited, often nonrenewable resource, it is critical that its use is carefully and transparently governed.

In some situations, excess stored diagnostic tissue may be accessed for research purposes. Indeed, as diagnostic tissue is linked to clinical data and outcomes, diagnostic tissue blocks offer a unique and valuable resource for translational and clinical research, including the evaluation of cancer biomarkers and drug-development studies.^2^ Tissue can also be obtained purely for research purposes, in which case it would be stored in a research biobank and governed by different rules. In both settings, the likelihood of patients being willing to undergo a major or minor procedure to allow the excision of additional tissue purely for research purposes is not well understood.

While patients have control over tissue whilst in their body, the act of excision can be seen as changing their relationship to that material. Similarly, when patients donate tissue for research purposes, they are often considered to have gifted their tissue, having been satisfied that the proposed uses and governance of that tissue are ethical and worthwhile.^2^ Nonetheless, there remains significant argument amongst patients, physicians, bioethicists, and researchers regarding the ownership of diagnostic tissue, particularly if knowledge or profit accrues from its use.^3-12^ For example, the argument remains strong regarding the utility and ethical acceptability of the outcome of the seminal US legal case of *Moore v. Regents of the University of California*, in which a patient whose excised cells had been used to create a commercially available immortal cell line lost their case for compensation.^13^ Indeed, while consistently rejecting the notion of patient ownership of diagnostic tissue, case law and statutory instruments globally have tended to leave this issue either undefined or unclear.^14^

Most jurisdictions generally enshrine ownership of excised diagnostic tissue in the entity that procures the test or stores the tissue, for example, a pathology department.^14^ In certain jurisdictions, property rights over tissue and body parts previously required “work or skill” to be applied to the sample, but recent legal decisions on some forms of tissue (some in Australia) have begun to recognize that such rights can be vested even without work or skill being applied.^14^ While it is not yet certain how the ongoing application of the “work or skill” legal test will apply to the types of tissue used in MP (and recognizing that this may differ from jurisdiction to jurisdiction), the uncertainty is likely to add to the complexity of this issue—not least because MP is becoming more widespread. In Australia, as elsewhere in the world, many of these issues remain unclear. Legal decisions in cases that have explored tissue property rights have usually been specific to particular types of tissue (for example, sperm) and may not generalize to other forms of tissue (such as biopsied tissue from a tumor). The degree of control a patient has over tissue ceded to others in a clinical or research setting remains ambiguous, with consent requirements differing from jurisdiction to jurisdiction.^15^

A key concern regarding property in tissue used in MP is what might happen if the expectations and understanding of patients and current custodians of tissue are not aligned. Studies that have explored consumer views of tissue ownership have tended to focus on tissue donated for research purposes to biobanks. These studies have found that consumers often do have concerns founded on cultural, religious, or ethical grounds about the future use of their donated tissue, and would like some control over decision-making in this context.^16-19^ Furthermore, Nicol et al^20^ found that a large section of the Australian population expects benefits (financial and health care) to accrue to individuals donating tissue to biobanks, in particular, affordable, universal health care. This appeared to arise out of a fairness principle. However, patient views and expectations regarding samples collected for diagnostic purposes (as in our study) may differ.

To understand how patients with cancer view the ownership and use of their excised tissue, as well as how they approach the hypothetical possibility of providing additional tissue purely for research, we conducted a mixed methods study of patients already participating in a research program that undertakes MP on diagnostic tissue for combined clinical and research purposes. We aimed, firstly, to determine patients’ preferences regarding ownership over, access to, and payment for diagnostic tissue, and how that matches with their understanding of regulation; and secondly, to determine the level of risk patients with cancer are willing to take to provide additional tissue for research purposes, and the demographic and disease factors associated with that willingness. We chose a mixed methods design to enable the exploration of both patient views in a large sample and perspectives about the same issues in more depth, in a smaller subsample.

## Methods

### Participants

Participants were recruited to a cancer genomic study, the Molecular Screening and Therapeutics (MoST) Program^21^ which is being conducted at the Garvan Institute of Medical Research in Sydney, Australia. MoST is recruiting adult patients with pathologically confirmed advanced or metastatic solid cancers of any histological type, who have exhausted therapeutic options. To be eligible, patients need sufficient accessible tissue for MP. MP on participants’ tissue is performed and, if an actionable variant is found, participants are enrolled in a related therapeutic trial if available. Participants were informed that while personal benefit was possible, the likelihood was low.

The Psychosocial Issues in Genomics in Oncology (PiGeOn) Project is a longitudinal, mixed methods psychosocial substudy of MoST which aims to examine the psychosocial and behavioral impacts of MP, and ethical issues involved in that process.^22^ Patients give written consent to this study at the same time as giving consent to the parent study. Both studies were approved by the St Vincent’s Hospital Human Research Ethics Committee (Reference number HREC/16/SVH/23).

### Data collection

While the PiGeOn study addresses a broad range of questions, this paper focuses on participants’ views regarding the ownership and use of tissue. All participants were asked to complete a questionnaire at the time consent was given (prior to MP), which included questions related to: whether participants would undergo excision of additional tissue for research purposes if no accessible tissue were available, if a minor, major or high-risk procedure was involved; views and knowledge regarding who owns and can access excised tissue for research use; and who should pay for any processing involved. Participants also completed measures of knowledge of genomics (the Knowledge of Genome Sequencing (KOGs) questionnaire^23^ comprising 9 items with higher scores reflecting better knowledge), and health literacy^24^ (3 questions assessing the need for help reading, problems reading and confidence with completing forms in the medical context, with higher scores reflecting lower health literacy).

For the qualitative substudy, a subgroup of patients was invited to participate in a semistructured interview of approximately 40 minutes duration. Purposive sampling was used to ensure heterogeneity in the sample. Those who agreed to participate were scheduled for a telephone interview at a time of their choosing, within 1-2 weeks of giving consent. Interviews were conducted by a trained qualitative researcher (S.V.) and continued until data saturation was reached. Interview questions further explored respondents’ questionnaire responses (Table 1). Questions asked and probes used to elicit information were developed iteratively as required to develop themes identified during the study analysis. Demographic details were collected by the parent study.

**Table 1.** Interview Schedule.

1. If genetic testing of the tumor were available to you and there was not enough tissue in storage, would you be willing to undergo a procedure to obtain tissue? 2. How much risk would you be willing to undertake to obtain the tissue? 3. What would influence your decision to have/not have a biopsy? 4. After you have an operation to remove all or part of your cancer, it is required by law that the pathology laboratory stores some of your tumor tissue for 7 years. Did you know this before you participated in the MoST program? 5. Who do you think OWNS your tumor tissue stored in the diagnostic pathology laboratory? 6. Who do you think SHOULD OWN your tumor tissue stored in the diagnostic pathology laboratory? 7. Who do you think SHOULD decide what happens to your tumor tissue that is stored in the diagnostic pathology laboratory? 8. Who do you think SHOULD PAY for preparation of a patient’s stored tumor tissue if it is being used for a clinical trial to guide treatment?

### Analysis

Demographic data were tabulated and summary statistics were used to describe survey results (Tables 2 and 3). Analysis of variables potentially associated with the desire to receive each type of result was performed using logistic regression using IBM SPSS Statistics Version 25. Variables investigated included age, sex, education, urban versus rural/remote place of residence (Accessibility and Remoteness Index of Australia [ARIA] as a proxy for socioeconomic status [SES]), Culturally and Linguistically Diverse [CALD], medical-science occupation, whether participants had biological children, whether first degree relatives were diagnosed with cancer, time since diagnosis, and cancer incidence, knowledge of genomics and health literacy (Table 4).

**Table 2.** Participant characteristics.

	Total sample (*n* = 356) *N* (%)
Sex	
Female	188 (52.8)
Male	168 (47.2)
Education level	
Primary/secondary school/vocational training	232 (65.2)
University	124 (34.8)
Accessibility and Remoteness Index of Australia	
Major city	261 (73.3)
Regional	89 (25.0)
Remote	5 (1.4)
Overseas	1 (0.3)
Medical/science occupation	
Yes	24 (6.7)
Parental status	
Yes	280 (78.7)
Speaks a language other than English at home	
Yes	73 (20.5)
Cancer incidence	
Common (>12 incidences/100,000 population)	113 (31.7)
Less common (6-12 incidences/100,000 population)	64 (18.0)
Rare (<6 incidences/100,000 population)	179 (50.3)
Multiple primary cancers	
Yes	60 (16.9)
First-degree relatives with cancer	
Yes	177 (49.7)
Previously attended family cancer clinic	
Yes	41 (11.5)
Previous genetic testing	
Yes	72 (20.2)
No	273 (76.7)
Don’t know	11 (3.1)
ECOG performance status	
0	132 (37.1)
1	209 (58.7)
2	15 (4.2)
Age at consent	
Mean (SD)	56.8 (13.9)
Median	59.0
Range	18-85
Time since first cancer diagnosis (months)	
Mean (SD)	43.5 (64.8)
Median	20.0
Range	0-498
Socioeconomic status index	
Mean (SD)	7 (3)
Median	7
Range	1-10
Charlson Comorbidity index	
Mean (SD)	2 (2)
Median	1
Range	0-9
Knowledge of genome sequencing (KOGS) score (%)	
Mean (SD)	64.6 (17.5)
Median	69.2
Range	0-100
Health literacy	
Mean (SD)	8.4 (3.1)
Median	9.0
Range	0-12

**Table 3.** Participant survey responses.

Survey questions	Participant responses (*n* = 356)		
	A minor procedure: a needle biopsy under a general anesthetic	A major procedure: an operation under general anesthetic	A high-risk procedure: an operation under general anesthetic with high risk of problems
	*n* (%)	*n* (%)	*n* (%)
If genetic testing of the tumor was available to you and there was not enough tissue in storage would you be willing to undergo?			
Yes	281 (79%)	162 (46%)	68 (19%)
No	24 (6.7%)	74 (21%)	137 (38%)
Unsure	49 (14%)	115 (32%)	147 (41%)
Missing	2 (0.6%)	5 (1.4%)	4 (1.1%)
Of the following factors, which would have the GREATEST influence on your decision to have a biopsy?			
Desire to contribute to research	11 (3.1%)		
The chance to guide my treatment	257 (72%)		
The chance to learn more about my cancer	20 (5.6%)		
The chance to predict the outcome of my cancer	25 (7.0%)		
Trust in my doctor	20 (5.6%)		
Other	21 (5.9%)		
Missing	2 (0.6%)		
Of the following factors, which would influence your decision the LEAST to have a biopsy?			
Desire to contribute to research	80 (22%)		
The chance to guide my treatment	16 (4.5%)		
The chance to learn more about my cancer	42 (12%)		
The chance to predict the outcome of my cancer	74 (21%)		
Trust in my doctor	84 (24%)		
Other	57 (16%)		
Missing	3 (0.8%)		
If you had decided NOT to have a biopsy to get enough tumor tissue for testing, which of the factors would be the GREATEST influence in your decision?			
Concerns about the privacy and confidentiality of genetic test results	1 (0.3%)		
The chance for a delay in my treatment while awaiting my genetic test results	76 (21%)		
The chance of a serious complication from the biopsy	211 (59%)		
The chance that the genetic test result could lead to insurance discrimination	3 (0.8%)		
The chance that the genetic test results would not help in finding a new treatment	38 (11%)		
Other	25 (7.0%)		
Missing	2 (0.6%)		
If you had decided NOT to have a biopsy to get enough tumor tissue for testing, which of the following factors would LEAST influence you in that decision?			
Concerns about the privacy and confidentiality of genetic test results	106 (30%)		
The chance for a delay in my treatment while awaiting my genetic test results	30 (8.4%)		
The chance of a serious complication from the biopsy	40 (11%)		
The chance that the genetic test result could lead to insurance discrimination	108 (30%)		
The chance that the genetic test results would not help in finding a new treatment	50 (14%)		
Other	19 (5.3%)		
Missing	3 (0.8%)		
After you have an operation to remove all or part of your cancer, it is required by law that the pathology laboratory stores some of your tumor tissue for 7 years. Did you know this before you participated in the MoST program?			
Yes	106 (30%)		
No	228 (64%)		
Unsure	22 (6.2%)		
Who do you think OWNS your tumor tissue stored in the diagnostic pathology laboratory?			
Doctor who obtained the tissue	12 (3.4%)		
Government	17 (5%)		
Pathology laboratory storing the tissue	91 (26%)		
You or your next of kin	122 (34%)		
No-one	9 (2.5%)		
Don’t know	96 (26%)		
Other	9 (2.5%)		
Who do you think SHOULD OWN your tumor tissue stored in the diagnostic pathology laboratory?			
Doctor who obtained the tissue	16 (4.5%)		
Government	7 (2.0%)		
Pathology laboratory storing the tissue	75 (21%)		
You or your next of kin	178 (50%)		
No-one	11 (3.1%)		
Don’t know	59 (17%)		
Other	10 (2.8%)		
Who do you think SHOULD decide what happens to your tumor tissue that is stored in the diagnostic pathology laboratory?			
Doctor who obtained the tissue	42 (12%)		
Government	7 (2.0%)		
Pathology laboratory storing the tissue	52 (15%)		
You or your next of kin	200 (56%)		
No-one	2 (0.6%)		
Don’t know	42 (12%)		
Other	10 (2.0%)		
Missing	1 (.3%)		
Who do you think SHOULD PAY for preparation of a patient’s stored tumor tissue if it is being used for a clinical trial to guide treatment?			
Doctor who obtained the tissue	3 (0.8%)		
Government	234 (66%)		
Pathology laboratory storing the tissue	15 (4.2%)		
You or your next of kin	21 (5.9%)		
No-one	6 (1.7%)		
Don’t know	43 (12%)		
Other	33 (9.3%)		
Missing	1 (0.3%)		

**Table 4.** Logistic regression analysis: level of risk and demographic/clinical characteristics (*N* = 356).

Explanatory variables	A minor procedure: a needle biopsy under a general anesthetic	A major procedure: an operation under general anesthetic	A high-risk procedure: an operation under general anesthetic with high risk of problems
	OR (95% CI)	OR (95% CI)	OR (95% CI)
Age	0.98 (0.96-1.00)*	0.98 (0.96-0.99)**	0.99 (0.97-1.02)
Education Level			
Primary or secondary education	Reference	Reference	Reference
University education	1.59 (0.84 -3.02)	0.69 (0.41-1.15)	0.74 (0.38-1.42)
Socioeconomic status	0.93 (0.84-1.03)	0.91 (0.84-0.99)*	0.91 (0.82-1.01)
Medical/Science occupation			
No	Reference	Reference	Reference
Yes	1.17 (0.31-4.37)	0.45 (0.17-1.18)	1.28 (0.41-3.98)
First-degree relative with cancer			
No	Reference	Reference	Reference
Yes	0.96 (0.56-1.65)	1.12 (0.72-1.75)	1.31 (0.75-2.28)
ECOG performance status	1.36 (0.84-2.22)	1.15 (0.77-1.71)	1.35 (0.82-2.21)
Previous genetic testing			
No	Reference	Reference	Reference
Yes	0.93 (0.45-1.93)	0.99 (0.55-1.76)	0.56 (0.26-1.23)
Knowledge of genome sequencing	1.02 (1.00-1.03)*	1.02 (1.00-1.03)*	1.01 (0.99-1.03)
Health literacy	1.04 (0.95-1.13)	1.09 (1.01-1.17)*	1.07 (0.97-1.18)
Model Goodness of Fit Test	Chi-square (9, *n* = 356) = 19.39	Chi-square (9, *n* = 356) = 26.51	Chi-square (9, *n* = 356) = 10.98
	*P* < .05	*P* < .01	*P* = .28

Abbreviations: ECOG, Eastern Cooperative Oncology Group; OR, Odds ratio.

*P < .05; **P < .01.

Interviews were recorded and transcribed. Using thematic analysis,^25^ relevant data were coded line-by-line by members of the research team. Initial codes from the transcripts were grouped to form focused codes which were applied to further transcripts. Using the constant comparative method, new codes were written as required over several meetings and collated into potential themes. Data collection and analysis occurred concurrently as themes were refined and applied to the data. Any differences between researchers (who had backgrounds in medicine, psychology, and bioethics) were resolved through discussion and negotiated consensus. Rigor was derived from successive discussions and review of the coding process by multiple authors until theoretical coding was complete. The varied academic backgrounds of the researchers ensured reflexivity, and the comparison of qualitative and quantitative results provided triangulation of data.

## Results

### Quantitative findings

Of 397 patients invited to join the PiGeOn study, 356 (90%), completed the questionnaire. Participants’ gender was broadly representative of the population (52.8% female). They had an average age of 56.8 years, and one-third (34.8%) had a university education (Table 2). Twenty percent spoke a language other than English at home. About half (50.3%) had a rare cancer (ie, a cancer with a population prevalence <6 per 100,000). Participants scored a mean of 64.6/100 on the Knowledge of Genomics Sequencing (KOGS) measure (17), and a mean of 8.4 on health literacy (18), indicating moderate knowledge and literacy.

Half of the participants (50%) believed they or their next of kin should own excised diagnostic tissue, with the next largest group (21%) believing the entity storing the tissue should own it (Table 3). Similarly, just over half (56%) believed they should control access to diagnostic tissue. Fewer (34%) believed they did own such tissue. Most participants (66%) believed the government (as the primary health funder in the study setting) should pay for tissue preparation and use for MP.

Patients’ willingness to undergo additional biopsies to obtain tissue for research purposes reduced as the complexity of the procedure increased, down from 79% willing to undergo a minor procedure such as a biopsy, to 46% for a major procedure involving an operation, and then to 19% for a major, high-risk operation (Table 3). Most participants (72%) stated that the major factor influencing their willingness to undergo a further biopsy was the possibility of finding something that could guide treatment. Conversely, most (59%) stated that concern about side effects would most influence their non-willingness, with concern about a possible delay to their treatment while awaiting a genetic result the second most common concern (21%). In a logistic regression (Table 4), age influenced both willingness to undergo a minor (OR = 0.98, 95% CI 0.96-1.0), or a major (OR = 0.98, 95% CI 0.96-0.99), procedure, with older patients less willing. Participants with greater knowledge of genomics were more willing to undergo a minor or major procedure (both, OR = 1.03, 95% CI 1.00-1.03). Those with higher health literacy (OR = 1.00, 95% CI 1.01-1.17) were more willing and those with higher SES (OR = 0.91, 95% CI 0.94-0.99) were less willing to undergo a major procedure. No demographic or disease factors were associated with willingness to undergo a major, high-risk operation.

### Qualitative findings

Twenty-three participants were interviewed (12 females and 11 males). Themes identified related to (1) a change from ownership to custodianship of tissue on removal from the body, (2) changing value of tissue across time and culture, (3) ethical issues underlying payment for tissue preparation and use, and (4) cost-benefit considerations in deciding on additional biopsies. Quotes underlying each theme are shown in Table 5.

**Table 5.** Participant quotes.

**From ownership to custodianship of tissue samples** *The body is a boundary* “Once they take it out of me, that’s it. I mean you can put it in the rubbish bin or you can put it in the—store it and use it. You know, it’s not mine anymore.”—K3067: Female, aged 63 “Just because my oncologist told me. Also, the research team… that I owned it. I had to give permission if it was to be used again.”—K3151: Female, aged 81 *Custodianship of samples* “I’m sure that it would be treated with respect… I don’t want it to be wasted… if it can be used, that’s the responsibility of the person who—or the hospital who has stored it.”—K3151: Female, aged 81 “The patient doesn’t have the sensitive skills or knowledge or capacity to really store it or own it… we would as patients divulge that responsibility to someone else when we give the sample to them… The government, because they set laws and regulations around various medical issues and probably passed legislation around that.”—K3194: Male, aged 35 *Graduations of consent* “I don’t think I’d really need to give permission. Maybe just a letter or something saying we’ve taken a portion of a sample and are doing this test on it and this is the outcome. I’d like to know the outcome.”—K3067: Female, aged 63 “You don’t want people having their samples and stuff tested without patient consent really. Because otherwise you end up in a sort of a murky situation.”—K3194: Male, aged 35 **The value of excised tissue changes over time and between cultures** “It is precious in the way it’s unique to me…, it would be the link in a research study.”—K3151: Female, aged 81 “I’m a nurse, and I know some cultures want an intact body to bury.”—K3067: Female, aged 63 **Ethical issues underlie views on who should pay** *Beneficiary pays* “If it was… going to directly benefit the patient well then that might be appropriate. If that was the only outcome of that work.”—K3194: Male, aged 35 *Equity of access* “There is no harm in asking, but obviously it depends on how well off the patient or patient’s family is, because some people don’t have a means to do that...”—K3165: Male, aged 26 *A community resource* “Because, research, that’s a benefit for society as a whole, I think government should be supporting research and supporting that development.”—K3154: Female, aged 21 “Well, we’re hoping that the government would pay for that. You know, it is about trying to keep Australian citizens alive, or worldwide citizens alive.”—K2970: Female, aged 66 *User pays* “If people want to use it for research, they should pay for it.”—K3151: Female, aged 81 “The research team, I guess. That obviously adds cost to research. But if they were wanting to do specific testing and the way that they store it might be more rigorous than usual.”—K3194: Male, aged 35 **Weighing up the pros and cons: Additional biopsies** *Benefits and risks* “Just knowing there could be a possibility that more information could [be found]—I’m willing to do that.”—K3154: Female, aged 21 “I’d need an awful lot of advantages, because to me, once it’s opened up, it’s open to the air and it would spread even further and quicker.”—K2986: Female, aged 73 “It would just drag things out worse and take up medical appointment time.”—K3040: Female, aged 51 *Weighing up* “(Regarding an invasive procedure): Not at this stage. I don’t think I could sustain it… I would certainly want to speak to my oncologist about it first.”—K3151: Female, aged 81 “I wouldn’t want to pull out more tissue unless it was absolutely necessary for me.”—K3165: Male, aged 26 “I’m comfortable with the risks associated with that because from my point of view the scientific interest is trumps. I know it sounds really crazy, but it trumps my own health.”—K3194: Male, aged 35

#### From ownership to custodianship of tissue samples

Participants had diverse views and understandings regarding who might own (ie, have complete control over) tissue samples and when that sample is deemed to be gifted/donated. They also differed in their understanding regarding custodianship of the sample (ie, the obligation of caretaking or safeguarding a sample on behalf of another, from initial collection to participation and/or consultation in decision-making around the storage and use of the sample).

##### The body is a boundary.

Some participants viewed the body as a boundary beyond which (once removed), tissue no longer belonged to the person from which it had come. They acknowledged a change in the form, function, and use of such tissue, once it left their body. Furthermore, they felt that in consenting to its removal, they had ceded responsibility for the sample over to those who had removed it.

A few participants felt that since the tissue came from them, they should be consulted about its use. Some participants also cited advice from their doctor or the research team that their consent would be sought for future use, reflecting a sense of choice that remains with patients (or their representatives) to determine what happens to the sample collected.

##### Custodianship of samples.

Some participants believed that once removed from their body, tissue was no longer their property (ie, they no longer owned it), but rather, custodianship of the sample would be the responsibility of those who could use it best (medical and research staff). Custodianship becomes a responsibility and ethical obligation of those seeking to store and use the samples. Participants felt medical and research staff had the expertise to decide how their tissue could be best used. They trusted these staff to treat their excised tissue with respect and to make ethical decisions regarding its use. Some participants felt the government, who contribute to relevant regulation, also has a role to play in ensuring the ethical use of excised tissue.

##### Gradations of consent.

After providing a sample, most participants did not want to be continuously contacted to provide consent to future uses of their collected material. However, if results from future studies had implications for their own health or the health of their family, they wished to be notified. Those few participants who felt greater ongoing custodianship over their tissue, however, believed their consent should be sought for each future use and that they should be informed of any outcomes.

#### The value of excised tissue changes over time and between cultures

Some participants felt that tissue within one’s body, if malfunctioning (as in cancer), is malicious and dangerous, and needs to be removed. Its removal has value to the individual. Once removed, the tissue no longer has a negative connotation and instead takes on the value of its potential uses (either for diagnosis or research). The information or knowledge generated from that sample has inherent value, potentially both to the individual from which it came and society at large.

Some participants noted that individuals from some cultures or religions may value excised tissue differently, requiring it to be returned to the body when they die so that body integrity is maintained at burial.

#### Ethical issues underlie views on who should pay

##### Beneficiary pays.

A few participants believed that if there was potential or actual benefit from the sample for the person who provided the sample, then they should pay for its storage and use.

##### Equity of access.

Many participants believed patients should pay for storage and use according to ability—if a patient can afford to pay, they should. But if they cannot, then the government/institution should pay, so that no disadvantage occurs.

##### A community resource.

Some felt that stored tissue is a community resource that has the potential to benefit everyone through research findings. Thus, its storage and use should be paid for by the government.

##### User pays.

A few participants believed that whoever considers the tissue useful and wants to use it (including researchers or medical institutions) should pay, particularly if the storage and preparation required for that use was different from the standard.

#### Risk-benefit considerations governed views on additional biopsies

Participants weighed up different considerations regarding whether to have an additional biopsy for research purposes.

##### Risks.

Participants feared complications, suffering, and trauma to their body from additional biopsies, citing personal experiences of such effects following previous biopsies and/or treatment. Some were concerned that undergoing another procedure would disturb the malignancy, potentially causing it to spread or change the environment of the body and aggravate their condition. Others felt there was simply a risk of no benefit and wasted time due to their advanced, terminal condition.

##### Benefits.

Participants were willing to risk surgery or a procedure if there was a potential benefit to themselves via results guiding treatment, improving quality of life, or even providing a cure. Some were comforted at the thought that, even if nothing of personal value came from this additional tissue because their illness was too advanced, they were giving back to science and society to help others.

##### Weighing up the pros and cons.

Participants weighed up potential benefits against potential risks, often based on their previous experience of procedures. Personal benefit was usually weighted more heavily than altruism. Most participants motivated by altruism felt that, if their own safety and comfort would be compromised, this would have to take priority over potential benefit to others. One person felt science trumped personal comfort. Participants trusted their clinicians to use their medical expertise in judging when additional biopsies would be clinically required.

## Discussion

In this study of patients undergoing MP as part of a research study, attitudes to ownership of excised tissue were diverse. Many participants addressed issues of ownership and responsibility in terms of custodianship. Custodianship in this context represented an ongoing relationship to the tissue extracted from one’s body, while ceding expertise for decision-making to another. Within this framework, there were diverse views on what the tissue represents, who is now responsible for storing samples, and the degree to which patients might wish to be included in decision-making about the sample. For example, some participants believed they or their next of kin should have a say in guiding subsequent use of their excised tissue, because it came from their body and could have implications for themselves or future generations. Similar results were obtained from Canadian patients with leukemia^26^ who had participated in a local biobank, most of whom wanted to be informed of results beyond the initial test indication either directly or via their doctor. However, some did not want to be continuously connected to their excised tissue sample. This view was influenced by a distaste for diseased tissue which they wanted out of their body, or was linked to potential cultural and religious conceptions of their body and associated tissues.

Nevertheless, many participants were happy to cede custodianship of their tissue to the clinicians, researchers, and institutions who had excised it, and who had the expertise and could be trusted to determine which future uses would add value to society. This view largely aligns with academic and legal perspectives. Some have suggested that community education and guidelines are needed to guide practice in this arena,^3^ and, where the samples are collected for research purposes, called for the inclusion of caretaking responsibility for tissue samples to start earlier when the research is being planned.^27^ The American Society of Clinical Oncology (ASCO) ethical framework for including research biopsies in cancer clinical trials, recommends providing a clear and upfront scientific rationale for the inclusion of biopsies in trials, including those destined for a biobank, as well as an analysis plan and safety protocol.^28^ While ideal, evolving clinical research projects and ongoing efforts to develop and integrate cancer genomics with clinical care may challenge feasibility.

Dry^2^ suggested that decisions regarding the appropriate use of diagnostic tissue blocks should be made by heads of hospital pathology departments, together with institutional leaders, ethics boards/committees, and legal counsel as needed. A recent US workshop to discuss the use of diagnostic tissue in research^29^ concluded that the designated staff at institutions (ie, pathology departments) should be custodians of patients’ tissue and should honor patients’ wishes, to the extent consistent with applicable laws and professional standards. In such cases, it should be ensured that, when expressing their wishes, patients are aware of any personal utility accrued in the intended research.

While many of our participants felt personal ownership over their diagnostic tissue (to some degree), most believed patients should not have to pay for storage and preparation of tissue for research purposes. They cited ethical issues of equity of access and community or personal benefit to justify payment by government or research bodies. Some would, however, be willing to pay some part of the cost, if it was within their means, if use of the sample would be of direct benefit to them. However, it should be noted that means testing in this setting may bring its own complexities and costs. King^30^ argues that while patient payments may allow valuable research that would not otherwise go forward to proceed, there is a risk that desperate patients will view paying for research as their only means to attain life-extending treatment, which would constitute coercion. Thus, a significant ethical overview would be required if this ever became an acceptable model of research funding.

While legal clarity remains elusive, in a practical sense ownership of human tissue removed as part of a diagnostic or therapeutic procedure conventionally resides within pathology services. In our personal experience (as clinicians and researchers in this area), pathology departments in Australia vary in charges to provide tissue for research purposes, ranging from AU$30 to AU$400. In contrast to the research use of tissue, routine clinical tissue access is funded by the Australian government through the Medical Benefits Scheme (MBS item 72860: AU$85). It is widely recognized that clinical trials provide clinical benefits to patients.^31^ In recognition of the clinical and systemic value of clinical trials, Australian government’s aim to integrate clinical trials into health care under the National Clinical Trials Governance Framework.^32^ Clinical trials depend on access to tissue (for example, to perform biomarker screening). Extension of government funding to tissue retrieval for publicly funded clinical trials would represent an important step toward the integration of clinical trials into the standard of care. However, payment systems are likely to vary from country to country.

Most of our participants were willing to undergo minimally invasive procedures to obtain additional tissue if required for research purposes, but their willingness sharply declined if an operation or high-risk procedure was required. Given that a steady increase in undertaking additional biopsies has been documented in clinical studies,^33^ this is important. Our participants trusted their health professionals to advise them when potential benefits outweighed the potential costs of additional biopsies. Transparent and ethical procedures are therefore required to ensure such advice is in patients’ best interests. The ASCO framework cited above states that consent forms for trials involving additional biopsies should clearly state that the biopsy is optional, explain the conditions for participation, potential risks, and benefits, and state that the biopsy will have no direct benefit to the participant.^28^ An audit of studies undertaken before the diffusion of the ASCO framework^34^ noted only a 39% adherence rate to the ASCO framework for studies involving additional biopsies, with only 70% of consent forms specifying biopsy-related risks. Hopefully, future audits will report higher compliance with the framework. Increasing the size of the tissue sample taken at the time of diagnosis may also be something for clinicians to consider in the future.

Limitations to this study include the small amount of information given to participants about the storage, preparation, and procedures for collecting and using diagnostic and research tissue. Some participants, despite having consented to undergo MP as part of a research study, were thinking about these issues for the first time, and wavered in their views over the discussion. Some participants may not have appreciated the difference between clinical treatments and clinical trials. On the other hand, our findings provide a novel insight into the views of patients with advanced cancer on these complex ethical issues in the research setting. This is a population subgroup with knowledge and interest in MP, and further research in more diverse populations is indicated, as well as in a clinical context.

## Conclusion

As diagnostic tissue becomes an increasingly valued resource for both clinical and research purposes, its ownership and conditions of use remain controversial topics. While most guidelines do not recognize patient ownership, they do encourage consideration of patient preferences and wishes. The involvement of consumers through structured public deliberation has been found to be feasible and helpful in informing institutional or regulatory policy for biobanks^35^ and may prove helpful in the context of diagnostic tissue also. Most of our participants were willing to cede custodianship rights of their diagnostic tissue to their medical institutions and staff and trusted them to make ethical decisions about its use. However, they did expect to be informed of results that were obtained through the use of their tissue, particularly if it was of relevance to them. Most were unwilling to accept more than a minor procedure to provide additional tissue if required for research, increasing the value of excess diagnostic tissue. This is particularly the case in instances where the research is of direct medical interest to the patient involved. Regardless of the intended purpose, respectful and considerate use of diagnostic tissue is expected by patients who provide it, and this should be woven into governance about its use.

## Data Availability

The data that support the findings of this study are available on request from the corresponding author. The data are not publicly available due to privacy and ethical restrictions.
